# Study on the Differences in Transcriptome and Metabolome of Pectoralis Major Muscles Between Jiangshan Black-Bone Chickens and Baier Buff Chickens

**DOI:** 10.3390/ani16121798

**Published:** 2026-06-10

**Authors:** Luoyi Zhu, Shiru Li, Ayong Zhao, Zhijun Wang

**Affiliations:** 1 Department of Ecology and Biological Resources, College of Agriculture and Biotechnology, Lishui University, Lishui 323000, China; 2 College of Animal Science and Technology, Zhejiang Agriculture and Forestry University, Hangzhou 311300, China; 3 College of Animal Sciences, Zhejiang University, Hangzhou 310058, China

**Keywords:** Jiangshan black-bone chicken, Baier buff chicken, pectoralis major muscle, transcriptome, metabolome

## Abstract

Jiangshan black-bone chicken is well known for its nutritional and health-promoting benefits, and the high melanin content in its muscles gives it a distinctive black appearance. Melanin possesses strong free radical scavenging ability, which may influence the antioxidant capacity and flavor characteristics of muscle tissue. Therefore, we conducted RNA sequencing and non-targeted liquid chromatography–mass spectrometry (LC-MS)-based metabolomics sequencing on the pectoralis major muscles of Jiangshan black-bone chickens and ordinary chickens (Baier buff chickens), to investigate differences in muscle metabolic regulation between two types of chickens. We detected 88 differentially expressed genes (DEGs) and 124 differential metabolites (DMs), identified enrichment in the “Oxidative phosphorylation”, “Glutathione metabolism”, and “Melanogenesis” pathways. As a result, genes *CHAC1*, *GSTA2*, *DCT*, *EDNRB*, *TYRP1*, *ATP5F1EP2* and metabolites “Adenosine diphosphate (ADP)”, “Phosphate (Pi)”, “Pyrophosphate (PPi)”, “Oxidized glutathione (GSSG)”, “Spermine”, contributed to differences in antioxidant capacity between the pectoralis major muscles of Jiangshan black-bone chickens and Baier buff chickens. Our results indicated that the Jiangshan black-bone chickens could generate more adenosine diphosphate (ADP), thereby enhancing glutathione metabolism and melanin synthesis, which may facilitate the removal of reactive oxygen species (ROS) in muscle tissue.

## Simple Summary

This study looked at why Jiangshan black-bone chickens are considered especially nutritious. We compared the breast muscle of black-bone chickens with that of conventional chickens. By examining their genes and metabolites, we found that black-bone chickens have a special ability to produce more energy molecules (ADP). This process helps them better manage natural oxidative stress by boosting important metabolic pathways, including those related to the black pigment (melanin).

## 1. Introduction

Chinese indigenous chickens exhibit marked diversity in meat quality and flavor, evidenced by up to 685 VOCs (Volatile Organic Compounds) and 27 breed-specific biomarkers [1]. In recent years, with growing consumer demand for superior poultry meat quality, the antioxidant capacity of indigenous breeds has emerged as a significant research focus [2]. Among these breeds, Jiangshan black-bone chicken and Baier buff chicken, two representative indigenous breeds from Zhejiang Province, exhibit marked differences in slaughter performance, meat quality, and flavor compound accumulation. Notably, regulatory divergences in key metabolic pathways (e.g., glycolysis, fatty acid oxidation, and the tricarboxylic acid cycle) within their pectoralis major muscle tissue likely directly influence sensory attributes and nutritional value [1,3,4].

The oxidative-reductive status of muscle is a key determinant of meat quality. Recent research highlights the central role of glutathione metabolism in maintaining cellular redox homeostasis. However, regulatory variations in this pathway across indigenous chicken breeds remain inadequately characterized [5,6]. Dietary supplementation with glutamine has been shown to alleviate oxidative stress and enhance glutathione peroxidase activity in the breast muscle of broilers [7]. Significantly, oxidative stress induces S-glutathionylation, which is a post-translational modification of the proteins in *E. coli*. This modification substantially alters the DNA-binding affinity of the target protein [8]. In chicken pectoralis major muscle tissue, glutathione metabolism likely similarly influences muscle cell function, although the precise regulatory mechanisms await elucidation.

Jiangshan black-bone chicken is renowned for its unique melanin deposition, characterized by deep black pigmentation in the skin, muscles, and bones systemically. This phenotype is strongly linked to the differential expression of melanogenesis-related genes [9,10]. Melanin itself possesses potent free radical scavenging ability, protecting cells from oxidative damage. Furthermore, oxidative phosphorylation, as the core process of cellular energy conversion, is hypothesized to influence muscle metabolic status and meat quality outcomes. Notably, given the substantial demand for tyrosine and energy substrates in melanin synthesis, the mitochondrial functional state of melanocytes represents a plausible intrinsic link between melanogenesis and energy metabolism that warrants further investigation [11,12].

Therefore, to investigate the differences in pectoralis major muscle between Jiangshan black-bone chicken and Baier buff chicken, this study employs an integrated transcriptomic and non-targeted liquid chromatograph mass spectrometer (LC-MS) metabolomic sequencing. We systematically reveal the coordinated regulatory network involving the oxidative phosphorylation, glutathione metabolism, and melanogenesis pathways within their pectoralis major muscle. This provides novel insights into the molecular mechanisms governing meat quality trait formation in indigenous chicken breeds. Our findings will contribute significantly to understanding the biochemical basis underlying the development of unique germplasm resources and offer both theoretical foundations and candidate molecular markers for high-quality chicken breeding programs.

## 2. Materials and Methods

### 2.1. Animal Materials

All chickens were reared under the same environmental and management conditions in floor pens within the same poultry facility. Feed and water were provided ad libitum throughout the experiment. Birds were not housed individually. To minimize environmental variation, both breeds were maintained under identical temperature, lighting, feeding, and husbandry conditions. In this study, each individual chicken was considered an experimental unit because the analyses (phenotypic measurements, transcriptomics, metabolomics, and histological assessments) were conducted on tissues collected from individual animals rather than pooled pen-level samples. The primary objective of this study was to investigate breed-associated molecular and phenotypic differences at the individual level.

Three hundred 360-day-old Jiangshan black-bone chickens (*n* = 6) and Baier buff chickens (*n* = 6) were selected. All animals were housed in environmentally controlled facilities with ad libitum access to food and water and were monitored daily by trained staff to minimize pain and distress. No unexpected adverse events occurred during the study. Pectoralis major muscle tissue samples were collected, immediately snap-frozen in liquid nitrogen, and subsequently stored at −80 °C. This experiment was conducted in accordance with the animal welfare guidelines approved by the Animal Health Committee of Zhejiang Agricultural and Forestry University (Hangzhou, China), under approval number ZAFUAC202401.

### 2.2. RNA Sequencing (RNA-Seq) and Quantitative Real-Time PCR (qPCR)

The RNA sequencing analysis of samples was performed by Majorbio Bio-Pharm Technology Co. Ltd. (Shanghai, China). Total RNA was extracted from the pectoralis major muscle samples, and RNA quality was subsequently assessed to ensure an OD260/280 ratio of 1.8–2.2, an RIN value greater than 6.5, and a total RNA amount exceeding 10 ng. Messenger RNA (mRNA) was then isolated and fragmented using poly(T) magnetic beads, followed by purification to generate the sequencing library. The clean reads from each sample were aligned to the chicken reference genome GRCg7b (Gallus_gallus, http://asia.ensembl.org/Gallus_gallus/Info/Index, accessed on 9 June 2025) using the HISAT2 tool (http://ccb.jhu.edu/software/hisat2/index.shtml, accessed on 9 June 2025)). We quantified transcript abundance through fragments per kilobase per million reads (FPKM) normalization. The DESeq2 (v 1.38.0) identified differentially expressed genes (DEGs) under stringent criteria: statistical significance (*p* < 0.05), |log_2_ fold change (FC)| ≥ 1 and false discovery rate (FDR) < 0.05.

cDNA was synthesized from total RNA extracted from pectoralis major muscle tissues using reverse transcription. All primers, designed with the NCBI database (https://www.ncbi.nlm.nih.gov/, accessed on 15 July 2025)), are provided in Supplementary Table S1. qPCR was run in six biological and technical replicates. The relative expression level was calculated using the 2^−ΔΔCt^ method [13].

### 2.3. LC-MS Metabolome

The LC-MS/MS analysis was performed using a Thermo Fisher Scientific Q Exactive HF-X (ThermoFisher, Dreieich, Germany) mass spectrometer coupled to a Vanquish UHPLC (ThermoFisher, Germany) system at Majorbio Bio-Pharm Technology Co., Ltd. (Shanghai, China). Peak detection, filtering, and alignment were processed using the XCMS package (v 3.12.0) in Rstudio (v 2023.12.1). Data normalization was achieved through total peak area scaling to eliminate systematic bias.

Orthogonal partial least squares-discriminant analysis (OPLS-DA) was implemented in RStudio using the ropls package (v 1.6.2). Model validity was assessed using a permutation test (*n* = 200) to guard against overfitting, and the predictive performance was evaluated via the Q^2^ (cum) value. Differential metabolites (DMs) screening criteria required both a variable importance in projection (VIP) score > 1 and *p* < 0.05.

### 2.4. GO and KEGG Annotation and Enrichment

Functional classification of profiled genes and metabolites was conducted through the Gene Ontology (GO) resource (http://geneontology.org), while Kyoto Encyclopedia of Genes and Genomes (KEGG) pathway mapping (http://www.kegg.jp, accessed on 12 September 2025) annotated biochemical relationships. Enrichment analysis was implemented in Python (v 3.9) using the scipy module (v1.0.0). Significantly enriched biological pathways were identified based on an FDR < 0.05 following Benjamini–Hochberg correction for multiple testing.

### 2.5. Statistical Analysis

Data are expressed as mean ± S.E.M. All statistical analyses were performed using RStudio (v 2023.12.1) and Python (v 3.9). Intergroup comparisons employed independent-samples *t*-tests with significance denoted as: * *p* < 0.05; ** *p* < 0.01.

## 3. Results

### 3.1. Differential Gene Expression in Pectoralis Major Muscle Between Jiangshan Black-Bone Chickens and Baier Buff Chickens

The raw sequencing reads generated in this study were deposited in the CNCB GSA database (accession number CRA044809). Sequencing yielded approximately 651.1 million raw reads, of which 632.3 million high-quality clean reads were retained after quality control (Supplementary Table S2). Subsequent alignment to the reference genome achieved mapping efficiencies ranging from 83.53% to 86.22% across all samples (Supplementary Table S2). We detected a total of 18,436 genes, comprising 17,807 known genes and 629 novel genes (Supplementary Table S3).

**Figure 1:**
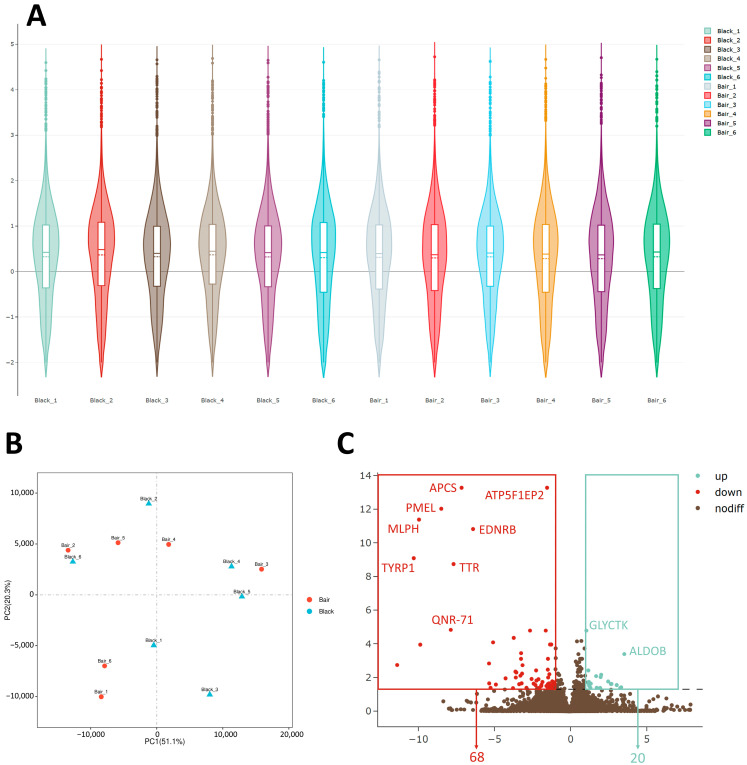
RNA sequencing information of Jiangshan black-bone chickens and Baier buff chickens. (**A**) Expression distribution of Jiangshan black-bone chickens and Baier buff chickens; (**B**) PCA shows the differences between Jiangshan black-bone chickens and Baier buff chickens; (**C**) Volcano plot displaying differentially expressed genes (DEGs) identified using DESeq2 (v 1.38.0). The horizontal dashed line indicates the significance threshold (FDR < 0.05), and vertical dashed lines demarcate the |log_2_ fold change| ≥ 1 cutoff.

Analysis of gene expression distributions revealed consistently high transcript levels with good stability across all samples (Figure 1A). Principal component analysis (PCA) demonstrated tight intra-group clustering and high inter-sample correlations, confirming the suitability of the data for downstream analyses (Figure 1B). Comparative transcriptomic analysis identified 88 DEGs between Jiangshan black-bone chickens and Baier buff chickens, with 20 genes upregulated and 68 downregulated in the Jiangshan black-bone chickens relative to Baier buff chickens (Figure 1C, Supplementary Table S4).

**Figure 2:**
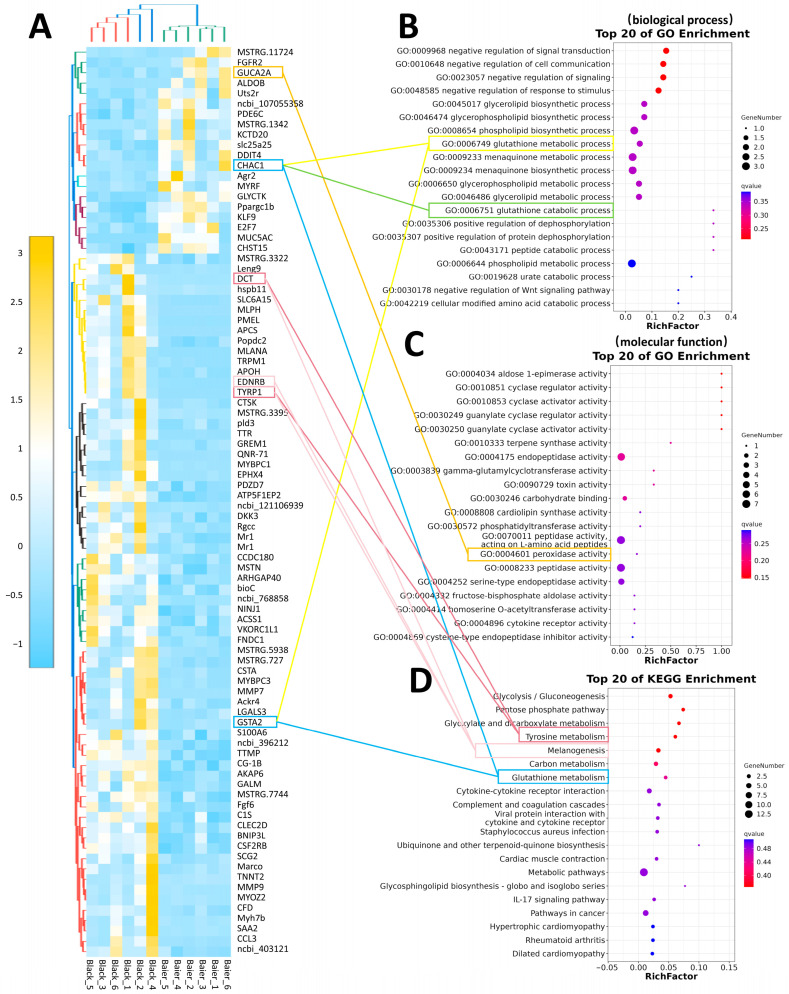
DEGs between Jiangshan black-bone chickens and Baier buff chickens. (**A**) Heatmap of DEGs expression in Jiangshan black-bone chickens and Baier buff chickens; (**B**) GO analysis of the biological processes of DEGs in Jiangshan black-bone chickens and Baier buff chickens; (**C**) GO analysis of the molecular functional of DEGs in Jiangshan black-bone chickens and Baier buff chickens; (**D**) KEGG analysis of DEGs in Jiangshan black-bone chickens and Baier buff chickens.

A heatmap visualization of DEGs is presented in Figure 2A. GO enrichment analysis revealed significant enrichments of biological processes in both breeds, particularly in “glutathione metabolic process” (*CHAC1* and *GSTA2*) and “glutathione catabolic process” (*CHAC1*) (Figure 2B, Supplementary Table S5). At the molecular function level, “peroxidase activity” (*GUCA2A*) was prominently enriched across the two chicken varieties (Figure 2C, Supplementary Table S5). These results indicate differences in antioxidant capacity between the pectoralis major muscles of the two breeds. Similarly, the two breeds were also enriched with the “Glutathione metabolism” (*CHAC1* and *GSTA2*) KEGG pathway (Figure 2D, Supplementary Table S6). In addition, melanin-related KEGG pathways “Melanogenesis” (*DCT*, *EDNRB* and *TYRP1*) and “Tyrosine metabolism” (*DCT* and *TYRP1*) were also enriched (Figure 2D, Supplementary Table S6). These results indicate a significant difference in melanin between the two pectoralis major muscles.

**Figure 3:**
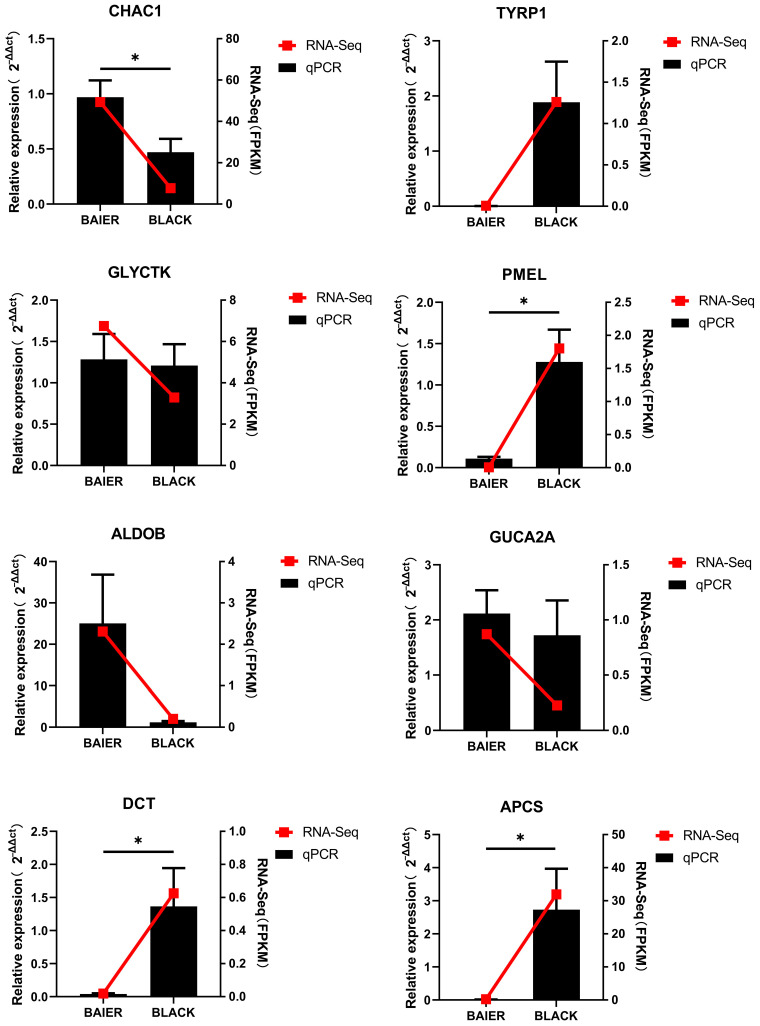
QRT-PCR verification of DEGs. Data were presented as means ± SEM. * *p* < 0.05.

To verify the authenticity of RNA sequencing, we selected 4 DEGs in the above items of concern (*CHAC1*, *TYRP1*, *GUCA2A* and *DCT*) and 4 DEGs with high expression levels (*GLYCTK*, *PMEL*, *ALDOB* and *APCS*) for qRT-PCR verification (Figure 3). The expression trends were consistent, indicating the confidence of the RNA sequencing results.

### 3.2. Differential Metabolite Expression in Pectoralis Major Muscle Between Jiangshan Black-Bone Chickens and Baier Buff Chickens

**Figure 4:**
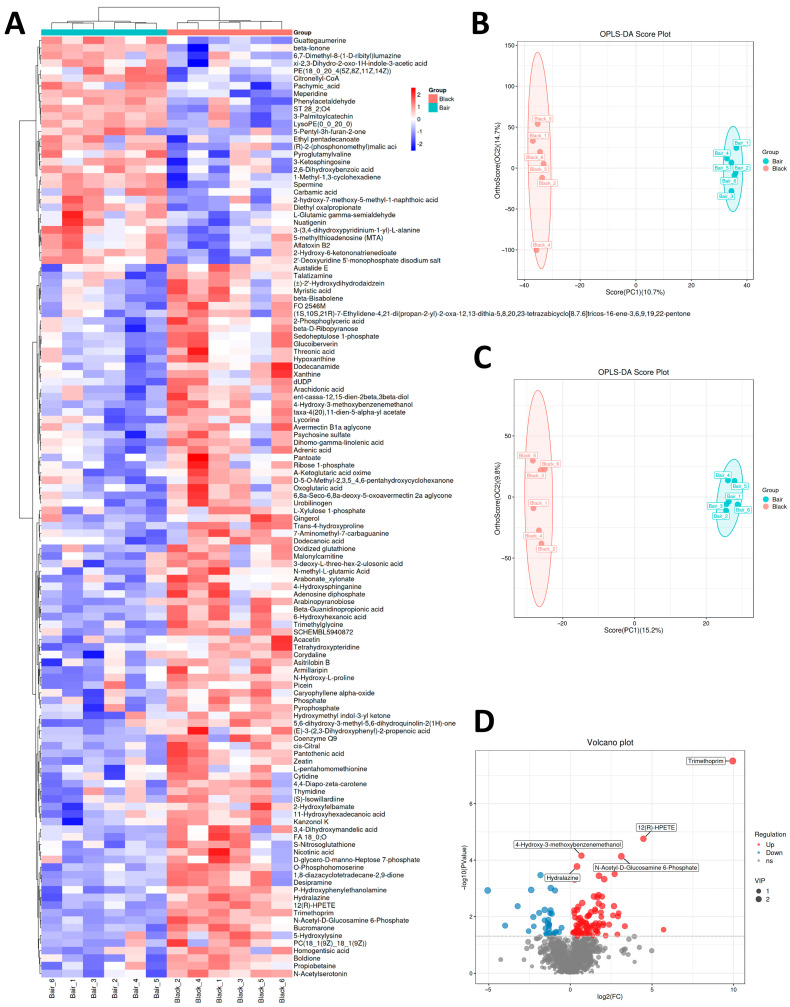
Differential metabolites (DMs) between Jiangshan black-bone chickens and Baier buff chickens. (**A**) OPLS-DA shows the differences in cationic metabolites between Jiangshan black-bone chickens and Baier buff chickens; (**B**) OPLS-DA shows the differences in anionic metabolites between Jiangshan black-bone chickens and Baier buff chickens; (**C**) GO analysis of the molecular functional of DEGs in Jiangshan black-bone chickens and Baier buff chickens; (**D**) Volcano plots of DMs between Jiangshan black-bone chickens and Baier buff chickens.

We employed a non-targeted LC-MS technique to study the differences in metabolite levels between the pectoralis major muscle tissues of the two breeds. The raw sequencing reads generated in this study were deposited in the CNCB OMIX database (accession number OMIX010882). We identified 12,385 positive ion metabolites and 5136 negative ion metabolites (Supplementary Table S7). OPLS-DA analysis of positive and negative ions data from both groups showed satisfactory within-group consistency and clear between-group separation (Figure 4B,C). Subsequently, OPLS-DA was used to analyze the overall differences in metabolic profiles between groups and to screen for DMs between groups using the criteria of VIP > 1 and *p* < 0.05 (Figure 4D). LC-MS identified 124 DMs between Jiangshan black-bone chickens and Baier buff chickens, with 94 up-regulated and 30 down-regulated in Jiangshan black-bone chickens relative to Baier buff chickens (Figure 4A, Supplementary Table S8).

**Figure 5:**
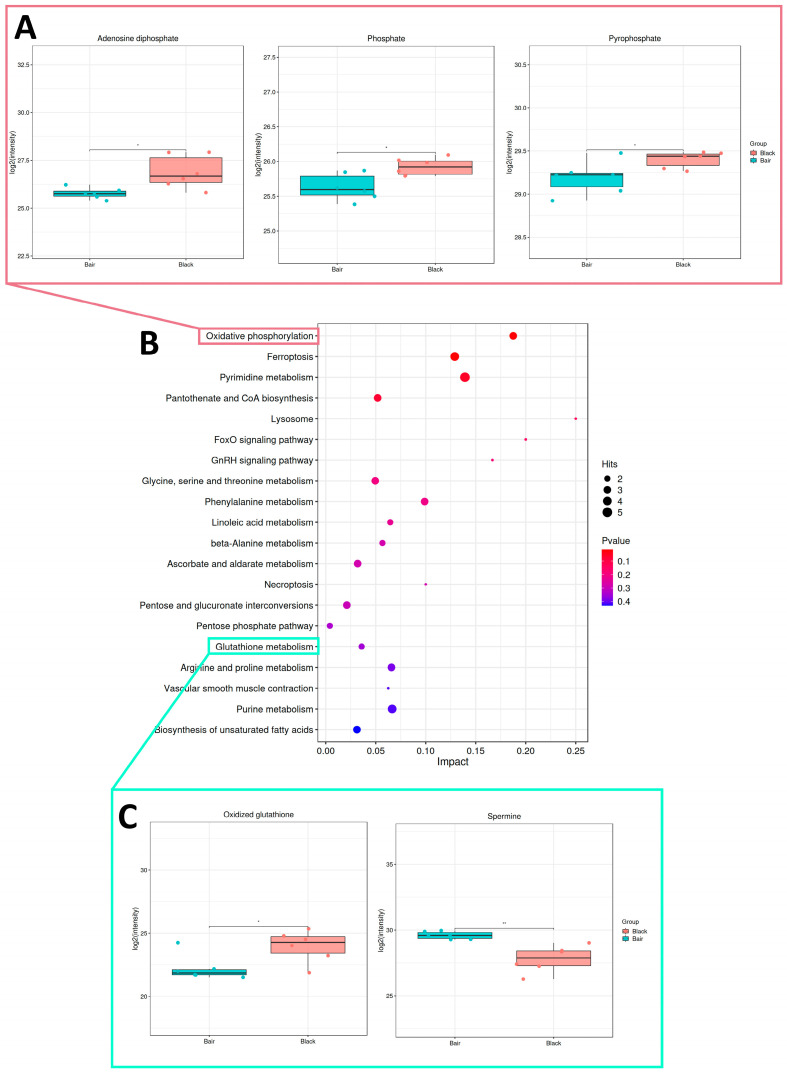
KEGG pathway analysis of DMs between Jiangshan black-bone chickens and Baier buff chickens. (**A**) DMs in the “Oxidative phosphorylation” pathway; (**B**) KEGG analysis of DMs in Jiangshan black-bone chickens and Baier buff chickens; (**C**) DMs in the “Glutathione metabolism” pathway. Intergroup comparisons employed independent-samples *t*-tests with significance de-noted as: * *p* < 0.05; ** *p* < 0.01.

KEGG analysis of DMs showed that the “Oxidative phosphorylation” and “Glutathione metabolism pathways” were significantly enriched between the two breeds (Figure 5B, Supplementary Table S9), and the DMs were “Adenosine diphosphate (ADP)”, “Phosphate (Pi)”, “Pyrophosphate (PPi)” (Figure 5A) and “Oxidized glutathione (GSSG)”, “Spermine” respectively (Figure 5C). These findings indicate metabolic differences in energy transfer and antioxidant capacity between the pectoralis major muscles of Jiangshan black-bone chickens and Baier buff chickens.

### 3.3. Integrated Analysis of DEGs and DMs in Pectoralis Major Muscle Between Jiangshan Black-Bone Chickens and Baier Buff Chickens

**Figure 6:**
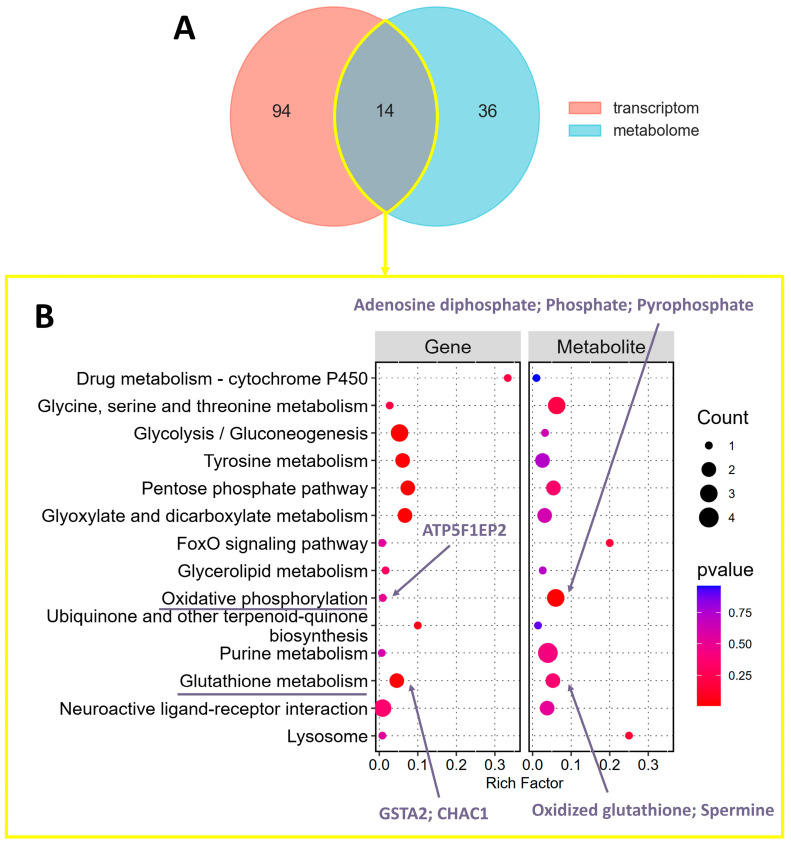
KEGG pathway analysis of DMs and DEGs between Jiangshan black-bone chickens and Baier buff chickens. (**A**) Venn Diagram shows common and unique KEGG pathway of DMs and DEGs between Jiangshan black-bone chickens and Baier buff chickens; (**B**) KEGG pathways of DMs and DEGs between Jiangshan black-bone chickens and Baier buff chickens.

It is difficult for a single omics to explain complex biological phenomena, so we correlated the results of the transcriptome and metabolome to explore the differences between the two types of chickens more convincingly. The transcriptome and metabolome were co-enriched in 14 KEGG pathways (Figure 6A). Notably, KEGG pathways “Oxidative phosphorylation (map00190)” (the DEG was *ATP5F1EP2,* whereas the DMs were “Adenosine diphosphate”, “Phosphate” and “Pyrophosphate”) and “Glutathione metabolism pathways (map00480)” (the DEGs were *GSTA2* and *CHAC1,* the DMs were “Oxidized glutathione” and “Spermine”) were simultaneously enriched (Figure 6B, Supplementary Table S10). In addition, KEGG pathway “Tyrosine metabolism (map00350)” (the DEGs were *DCT* and *TYRP1,* the DMs were “Homogentisic acid” and “3,4-Dihydroxymandelic acid”) was enriched (Supplementary Table S10). This means that these DEGs and DMs are key regulators of the differences between the two pectoralis major muscles.

**Figure 7:**
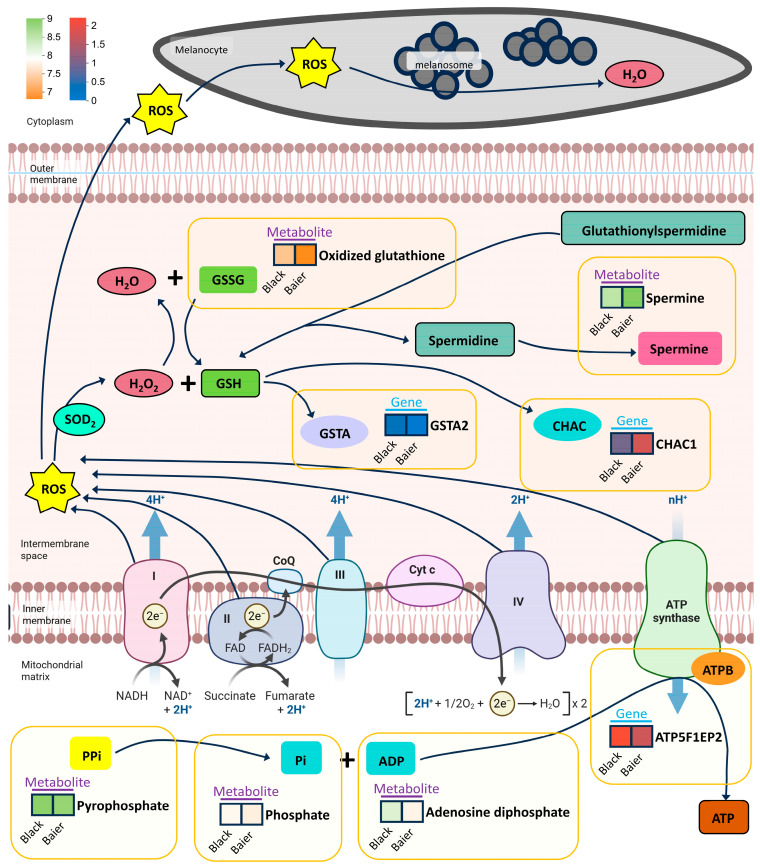
The regulatory network of pectoralis major muscle between Jiangshan black-bone chickens and Baier buff chickens.

The genes *GSTA2* and *CHAC1*, together with metabolites GSSG and spermine, participate in redox regulation associated with the electron transfer chain, clearing ROS and protecting mitochondrial membranes and enzyme complexes from oxidative damage. The gene ATP5F1EP2, along with metabolites PPi, Pi, and ADP, together form a cycling system for energy currency, utilizing the proton gradient generated by the respiratory chain to store and release energy. In Figure 7, the electron transport chain complexes (I–IV) drive adenosine triphosphate (ATP) synthesis by ATP synthase. ATP5F1EP2 may be involved in proton channel, compelling ADP and the Pi derived from PPi hydrolysis to form the high-energy phosphoanhydride bond of ATP. Reactive oxygen species (ROS) are generated during this process. Glutathione (GSH) neutralizes ROS, forming GSSG. Within this context, GSTA2 mediates detoxification through GSH-catalyzed conjugation reactions. *CHAC1* degrades excess GSH. Spermine indirectly modulates GSH levels. Furthermore, ROS can also be transferred to melanocytes for clearance via melanosomes. Among them, Jiangshan black-bone chickens exhibited higher expression levels of PPi, Pi, ADP, *ATP5F1EP2*, and *GSTA2* compared to Baier buff chickens (Figure 7). Conversely, Jiangshan black-bone chickens showed lower expression levels of *GSSG*, *CHAC1*, and spermine than Baier buff chickens (Figure 7). These findings suggest that Jiangshan black-bone chickens may catalyze ATP synthesis and antioxidant reactions more efficiently, resulting in better energy metabolism and stress resistance than Baier buff chickens, which may contribute to stronger muscle function and improved stress resistance.

## 4. Discussion

Jiangshan black-bone chickens, as a high-quality breed of black-bone chickens, have a higher eviscerating rate and inosine monophosphate (IMP) content than Baier buff chickens, resulting in better meat production performance and stronger meat flavor [1,14]. The transcriptome and metabolome results revealed distinct pathway activities in the pectoralis major muscle of Jiangshan black-bone chickens compared with Baier buff chickens. Specifically, these differences were observed in the “Oxidative phosphorylation (map00190)”, “Glutathione metabolism pathways (map00480)”, and “Melanogenesis (map04916)” pathways. Key regulatory genes (*CHAC1*, *GSTA2*, *DCT*, *EDNRB*, *TYRP1*, and *ATP5F1EP2*) and metabolites (Adenosine diphosphate, Phosphate, Pyrophosphate, Oxidized glutathione, and Spermine) were identified as drivers of these differences (Figure 2D, Figure 5B and Figure 6B).

The oxidative phosphorylation pathway, localized to the inner mitochondrial membrane, couples electron transport chain-mediated oxidation NADH/FADH2 with phosphorylation of ADP to generate ATP, serving as the core cellular energy transduction mechanism. The glutathione metabolic pathway, centered on reduced GSH, forms a critical cellular antioxidant defense system, utilizing its thiol group (-SH) to scavenge free radicals, detoxify heavy metals, and maintain redox homeostasis [15,16]. The melanogenesis pathway, initiated by tyrosinase, catalyzes the conversion of tyrosine to eumelanin or pheomelanin via dopaquinone, contributing to protection against UV radiation and oxidative stress [10].

We propose an integrated metabolic relationship among these pathways in Figure 7. On the inner mitochondrial membrane, oxidative phosphorylation facilitates energy transduction. Electron transfer through complexes I-IV drives protons (H+) pumping from the matrix to the intermembrane space, establishing an electrochemical gradient across the membrane. These gradient powers the rotational catalysis of ATP synthase. Within this complex, the *ATP5F1EP2* (ATP Synthase F1 Subunit Epsilon Pseudogene 2)-containing F_0_ subunit facilitates proton translocation, driving conformational changes in the F_1_ catalytic domain that synthesizes ATP from ADP and inorganic phosphate (Pi). The hydrolysis of newly synthesized ATP releases energy, regenerating ADP and Pi. Pyrophosphate (PPi), a byproduct of reactions such as nucleic acid synthesis, is hydrolyzed to Pi by mitochondrial alkaline phosphatase. Crucially, both electron transport and ATP synthesis generate reactive oxygen species (ROS), thereby imposing oxidative stress.

Concurrently, the glutathione metabolism pathway maintains cellular redox homeostasis through GSH synthesis, transformation, and degradation. GSH acts as a primary antioxidant by neutralizing ROS to form oxidized glutathione (GSSG) [17]. Glutathione reductase (GSR) regenerates GSH from GSSG, completing a redox cycle. *GSTA2* (Glutathione S-transferase A2) catalyzes GSH conjugation to electrophilic toxins, facilitating their detoxification and excretion. GSSG accumulation serves as an oxidative stress biomarker and can modulate signaling pathways and induce antioxidant gene expression. *CHAC1* (ChaC Glutathione Specific Gamma-Glutamylcyclotransferase 1) degrades cytoplasmic GSH [18]. Spermine, a polyamine, can indirectly regulate GSH levels, potentially by binding GSH or inhibiting *CHAC1* activity, thereby suppressing degradation and enhancing antioxidant capacity [19]. Additionally, melanosomes may participate in ROS clearance.

Our data indicate a significant enhancement of oxidative phosphorylation in the pectoralis major muscle of Jiangshan black-bone chicken, evidenced by elevated expression of the critical gene *ATP5F1EP2* and higher concentrations of metabolites ADP, Pi, and PPi compared to Baier buff chickens (Figure 7). This finding reflects increased mitochondrial electron transport chain activity. *ATP5F1EP2*, as an essential ATP synthase subunit, directly participates in proton translocation driving ATP synthesis. Supporting this inherent energetic advantage in black-bone breeds, studies in Xichuan black-bone chicks also show enriched oxidative phosphorylation and upregulated mitochondrial biogenesis genes. This advantage may stem from: (1) Higher muscle metal ion content (e.g., iron ions as cytochrome c oxidase cofactors facilitating terminal electron transfer); and (2) Characteristic melanin deposition potentially optimizing mitochondrial enzyme activity via metal ion chelation.

Furthermore, key alterations in the glutathione pathway were observed: significant upregulation of *GSTA2* and downregulation of the catabolic enzyme *CHAC1*, alongside reduced *GSSG* accumulation (Figure 7). This reveals the molecular basis for the enhanced antioxidant defense in Jiangshan black-bone chickens. *GSTA2* catalyzes GSH-dependent detoxification and ROS scavenging, mitigating oxidative damage [20].

Finally, melanin deposition in Jiangshan black-bone chickens confers not only phenotypic distinctiveness but also direct antioxidant activity. Indole-5,6-quinone structures within melanin effectively quench superoxide anions (O_2_^−^) and hydroxyl radicals (·OH). Melanogenesis requires substantial consumption of tyrosine and ATP consumption. Critically, the enhanced oxidative phosphorylation pathway identified here provides the necessary ATP, enabling Jiangshan black-bone chicken mitochondria to meet the energy demands of melanin synthesis—thereby amplifying the overall ROS scavenging capacity of the organism.

Although the sample size in the present study was relatively small, strict control of genetic background, age, and rearing conditions helped reduce environmental variability among individuals. Future studies with larger sample sizes will be necessary to further validate the findings.

## 5. Conclusions

Key regulatory genes (*CHAC1*, *GSTA2*, *DCT*, *EDNRB*, *TYRP1*, *ATP5F1EP2*) and metabolites (Adenosine diphosphate, Phosphate, Pyrophosphate, Oxidized glutathione, Spermine) underpin the differential antioxidant capacity observed in the pectoralis major muscle between Jiangshan black-bone chickens and Baier buff chickens. The enhanced oxidative phosphorylation activity in Jiangshan black-bone chickens facilitates greater ATP production. This energetic advantage subsequently augments glutathione metabolism and melanogenesis, synergistically promoting efficient ROS scavenging and mitigating oxidative stress within the muscle tissue.

## Data Availability

The original contributions presented in this study are included in the article/Supplementary Materials. Further inquiries can be directed to the corresponding author.

## Supplementary Materials

The following supporting information can be downloaded at: https://www.mdpi.com/article/10.3390/ani16121798/s1, Supplementary Table S1: The primers used for qRT-PCR; Supplementary Table S2: The information of RNA sequencing in each sample; Supplementary Table S3: Detected genes between two groups; Supplementary Table S4: Details of all DEGs between two groups; Supplementary Table S5: Enriched GO terms of DEGs between two groups; Supplementary Table S6: Enriched KEGG of DEGs between two groups; Supplementary Table S7: Detected metabolites between two groups; Supplementary Table S8: Details of all DMs between two groups; Supplementary Table S9: Enriched KEGG of DMs between two groups; Supplementary Table S10: Enriched KEGG of DEGs & DMs between two groups.

## References

[1] Li D., Zhang X.W., Yuan Q.Y., Zhou Z.X., Zhao A.Y. (2023). Comparative study on slaughter performance, meat quality and expression of inosinic acid-related genes in seven local chicken breeds. Chin. J. Anim. Sci..

[2] Dong S., Li L., Hao F., Fang Z., Zhong R., Wu J., Fang X. (2024). Improving quality of poultry and its meat products with probiotics, prebiotics, and phytoextracts. Poult. Sci..

[3] Cai Z.G. (2021). The Consideration on Protection and Utilization of Animal and Poultry Germplasm Resources in City Jiangshan. Zhejiang J. Anim. Sci. Vet. Med..

[4] Ding X.Y., Li S.R., You X.R., Du Y., Du X., Zhao A.Y., Wang Z.J. (2025). Differences of Thoracic Volatile Metabolites in Breast Muscle Between Jiangshan Black-bone Chicken and Baier Buff Chicken. China Poult..

[5] Gasmi A., Nasreen A., Lenchyk L., Lysiuk R., Peana M., Shapovalova N., Piscopo S., Komisarenko M., Shanaida M., Smetanina K. (2024). An Update on Glutathione’s Biosynthesis, Metabolism, Functions, and Medicinal Purposes. Curr. Med. Chem..

[6] Howie J., Tulloch L.B., Brown E., Reilly L., Ashford F.B., Kennedy J., Wypijewski K.J., Aughton K.L., Mak J.K.C., Shattock M.J. (2024). Glutathione-dependent depalmitoylation of phospholemman by peroxiredoxin 6. Cell Rep..

[7] Hu H., Chen L., Dai S., Li J., Bai X. (2020). Effect of Glutamine on Antioxidant Capacity and Lipid Peroxidation in the Breast Muscle of Heat-stressed Broilers via Antioxidant Genes and HSP70 Pathway. Animals.

[8] Wang T., Liu H., Liu H., Xia Y., Xun L. (2025). Oxidants induce Escherichia coli MarR glutathionylation in the presence of glutathione. Redox Biol..

[9] Tu Y.G., Xie M.Y., Sun Y.Z., Tian Y.G. (2009). Structural characterization of melanin from Black-bone silky fowl (Gallus gallus domesticus Brisson). Pigment Cell Melanoma Res..

[10] Zhou S., Zeng H., Huang J., Lei L., Tong X., Li S., Zhou Y., Guo H., Khan M., Luo L. (2021). Epigenetic regulation of melanogenesis. Ageing Res. Rev..

[11] Herrera A.S., Del C.A.E.M., Md Ashraf G., Zamyatnin A.A., Aliev G. (2015). Beyond mitochondria, what would be the energy source of the cell?. Cent. Nerv. Syst. Agents Med. Chem..

[12] Snyman M., Walsdorf R.E., Wix S.N., Gill J.G. (2024). The metabolism of melanin synthesis-From melanocytes to melanoma. Pigment Cell Melanoma Res..

[13] Livak K.J., Schmittgen T.D. (2001). Analysis of relative gene expression data using real-time quantitative PCR and the 2(-Delta Delta C(T)) Method. Methods.

[14] Li D., Wang X., Fu Y., Zhang C., Cao Y., Wang J., Zhang Y., Li Y., Chen Y., Li Z. (2019). Transcriptome Analysis of the Breast Muscle of Xichuan Black-Bone Chickens Under Tyrosine Supplementation Revealed the Mechanism of Tyrosine-Induced Melanin Deposition. Front. Genet..

[15] Handy D.E., Loscalzo J. (2022). The role of glutathione peroxidase-1 in health and disease. Free Radic. Biol. Med..

[16] Iskusnykh I.Y., Zakharova A.A., Pathak D. (2022). Glutathione in Brain Disorders and Aging. Molecules.

[17] Averill-Bates D.A. (2023). The antioxidant glutathione. Vitam. Horm..

[18] Wang Y., Niu H., Li L., Han J., Liu Z., Chu M., Sha X., Zhao J. (2023). Anti-CHAC1 exosomes for nose-to-brain delivery of miR-760-3p in cerebral ischemia/reperfusion injury mice inhibiting neuron ferroptosis. J. Nanobiotechnol..

[19] Tse R.T., Wong C.Y., Chiu P.K., Ng C.F. (2022). The Potential Role of Spermine and Its Acetylated Derivative in Human Malignancies. Int. J. Mol. Sci..

[20] Deng J., Zhao L., Zhang N.Y., Karrow N.A., Krumm C.S., Qi D.S., Sun L.H. (2018). Aflatoxin B(1) metabolism: Regulation by phase I and II metabolizing enzymes and chemoprotective agents. Mutat. Res. Rev. Mutat. Res..

